# 45724 VC2 Oncolytic Virotherapy Induces Robust Systemic Anti-Tumor Immunity and Increases Survival in an Immunocompetent B16F10-derived Mouse Melanoma Model

**DOI:** 10.1017/cts.2021.437

**Published:** 2021-03-30

**Authors:** Ifeanyi Kingsley Uche, Natalie Fowlkes, Luan Vu, Tatiane Watanabe, Mariano Carossino, Rafiq Nabi, Fabio Del Piero, Jared S. Rudd, Konstantin G. Kousoulas, Paul J.F. Rider

**Affiliations:** 1 Louisiana State University School of Veterinary Medicine; 2 The University of Texas MD Anderson Cancer Center; 3 North Carolina State University

## Abstract

ABSTRACT IMPACT: Our data demonstrate that VC2 oncolytic virotherapy has significant clinical potential. OBJECTIVES/GOALS: Use our novel oncolytic herpes simplex virus type I (HSV-1), VC2, to understand how oncolytic virotherapy affects the immunosuppressive tumor microenvironment as a mechanism of efficacy. METHODS/STUDY POPULATION: We tested the efficacy of VC2 as an oncolytic virotherapy (OVT) in a syngeneic B16F10-derived mouse model of melanoma. We modified the B16F10 to express nectin-1 (B16F10n-1), the major receptor for HSV-1. Engrafted B16F10n-1 tumors were intratumorally treated with either phosphate-buffered saline (PBS) or 1x10^6 pfu VC2. At indicated time points, treated tumors were excised and processed for immunohistochemistry or flow cytometry analysis. For our experimental metastasis studies, mice were intravenously challenged with B16F10n-1 cells. For our depletion studies, CD4+ and CD8+ T cells were depleted in mice by treatment with mouse anti-CD4 and anti-CD8 monoclonal antibodies respectively, while the control mice were given Rat IgG2b isotype. RESULTS/ANTICIPATED RESULTS: We found that VC2 slowed tumor growth rates and significantly enhanced survival times over control treated mice. VC2-treated mice that survived initial tumor engraftment were able to reject a second tumor challenge and were also resistant to lung colonization (experimental metastasis) of tumor cells. Furthermore, VC2 treatment promoted increased intratumoral T cell infiltration and induced a strong antitumor effect that decreased growth rates of distant, untreated tumors. DISCUSSION/SIGNIFICANCE OF FINDINGS: Our data demonstrate that VC2 OVT has significant clinical potential. Furthermore, due to the increased survival rates and CD8+ T cells dependence, our model will enable study of the immunological correlates of protection for VC2 OVT and OVT in general, as well as to inform the rational design of future OVs with improved therapeutic potentials.

